# Current Levels of Salt Knowledge: A Review of the Literature

**DOI:** 10.3390/nu6125534

**Published:** 2014-12-01

**Authors:** Rani Sarmugam, Anthony Worsley

**Affiliations:** 1 Health Promotion Board, 168937 Singapore, Singapore; 2 School of Exercise and Nutrition Sciences, Deakin University, Burwood VIC 3125, Australia; E-Mail: anthony.worsley@deakin.edu.au

**Keywords:** salt, sodium, knowledge, awareness, review, salt use, salt intake, salt practices

## Abstract

High salt intake increases the risk of hypertension and cardiovascular diseases. Given the role of knowledge as a determinant of food intake, this paper aims to review the current levels of salt knowledge and the association between salt knowledge and dietary salt intake and salt-related dietary practices in the general population. Twenty two studies were included in the review. In general, the studies showed consumers were able to identify the health risks associated with high salt intake. However, knowledge of recommended daily intakes, understanding of the relationships between salt and sodium and foods that contribute most salt to the diet were poor. Four of the five studies which examined the relationships between salt knowledge and salt-related dietary practices reported significant associations. Two important gaps in the current literature were identified. First, there is a need for a robustly validated tool to examine salt knowledge and its impact on salt intake. Second, a comprehensive salt knowledge assessment should include assessment of procedural, as well as declarative, knowledge.

## 1. Introduction

A large body of literature shows that high salt intake increases the risk of hypertension [1,2], cardiovascular diseases [3,4] and stroke [5,6]. Population salt intakes in most countries are above recommended levels [7]. The majority of salt in Western diets comes from processed foods [8,9,10], while in developing countries the majority of dietary salt is added to food during food preparation [7].

Several countries have voluntary salt reduction programs for food manufacturers to reduce the salt content of processed foods [11]. Although this approach has been shown to be a cost-effective way to reduce salt in the diet [12], it may take time and may vary within and between countries [13,14,15,16]. Therefore, until there is widespread availability of food products with lower salt contents, active participation from consumers is required to ensure that their salt intake is within the recommended levels. Consumer education and awareness are also particularly important in countries where the majority of salt is added in cooking and at the table.

Two types of knowledge are required for consumers to make informed choices [17,18]. These are: (1) declarative knowledge, also known as knowledge of “what is” (*i.e.*, awareness of things and processes) or “know that” knowledge (for example, consumers need to know the recommended level of salt intake and the health risks of high salt intakes); and (2) procedural knowledge, or “know how” knowledge. Procedural knowledge is about practical skills-how to carry out certain tasks. For example, how to choose a lower salt product by comparing food label information and how to reduce the amount of salt used in the cooking by using herbs and spices.

Given the importance of consumers’ role in salt reduction, and the possible influence of salt knowledge on salt consumption, this paper aims to review (1) the current levels of salt knowledge in the population; and (2) the relationships between salt knowledge and salt intake and salt-related dietary practices such as use of salt at the table, in cooking and the purchase of low/reduced salt products. Such understanding is important for the development of effective consumer education and awareness campaigns.

## 2. Experimental Section

### 2.1. Search Strategy

A systematic search was conducted on Scopus and Web of Science databases using the keywords: salt, sodium, nutrition, food, knowledge, awareness and combinations of these terms such as “salt knowledge” in article titles, abstracts and keywords for original research papers published from January 1990 to June 2014. The search results were screened based on titles and abstracts, and when in doubt, full articles were reviewed. A manual search of bibliographies of articles included in the review was conducted to ensure a more wide-ranging search.

Because of the small number of original research papers, a decision was made to include reports of consumer surveys published by governmental agencies and reputable organizations, such as Consensus Action on Salt and Health (CASH), found during the search of the bibliographies to provide a comprehensive view of the current status of salt knowledge in the population.

#### 2.1.1. Inclusion Criteria

Only studies published in the English language that assessed salt knowledge using quantitative methods in healthy adult populations were included.

#### 2.1.2. Exclusion Criteria

Studies that assessed salt knowledge among patients, health care professionals and food service operators, exclusively were excluded. Those that used qualitative methods, such as focus group studies, were also excluded.

### 2.2. Data Extraction

Data extracted from the papers or reports included details of the survey methods, study population and sample size. Brief descriptions of the study populations were also included.

The salt knowledge reported in the reviewed studies was classified into two broad categories; declarative knowledge and procedural knowledge. Although there were similarities between the areas of declarative knowledge, there were variations in the framing of the questions asked of respondents. Therefore, the results were organized into four themes. These were: (1) understanding of the relationships between salt and sodium; (2) recommended daily salt intake; (3) diet-disease relationships; and (4) salt content of commonly consumed foods. These themes are discussed below.

## 3. Results

### 3.1. Description of Studies

Twenty two studies were included in the review (see Table 1). Out of these, five were surveys commissioned by national health agencies or non-governmental organizations. Most of the studies were conducted in developed countries, mainly Australia, Canada, the USA, and the UK. Only three studies included populations in developing countries [19,20,21].

Half of the studies had more than 1000 participants. Not all papers explicitly described the survey sampling methods. Among those that did, seven used sampling frames that represented the national population. Among the studies that clearly indicated how the survey questions were administered, online administration was the most commonly used method (8 out of 22 studies).

#### Socio-Demographic Characteristics of Study Samples

With the exception of one study [22], all the studies in the review were conducted among participants within a wide age range (18 and above years). Eight studies had more than 60% women respondents. Of these, three studies were conducted only among women [23,24,25]. In six out of the 15 studies that provided information about participant’s education levels, at least 75% of the participants had completed high school education.

**Table 1:** Description of studies included in the review.

Author (Year)	Country (Representative Sample (Y/N))	Survey Method	Source of Questions	Description of Sample		
				*N* (% Female)	Age Range (Mean)	Education (% above High School)
Arcand *et al.*, (2009) [23]	Canada (Y) ^¶,§^	Online survey	Developed by sodium and/or consumer survey experts, or taken from similar national surveys	2603 (65.2) †	20–69	81.1 ^†^
Charlton *et al.*, (2010) [24]	Wollongong, Australia (N)	Self-administered	Adapted from past survey [26]	78 (100)	19–56 (38.3)	88
Claro *et al.*, (2012) [19]	Argentina, Canada, Chile, Costa Rica, and Ecuador (N)	Intercept survey	Self-developed using expert opinion of the expertise involved and past surveys	All countries: 1992 (55.9), Argentina: 400 (58.3); Canada: 399 (60.9); Chile: 400 (51.5); Costa Rica: 396 (51.5); Ecuador: 397 (57.4)	≥18	38.8
Consensus Action on Salt and Health (2003) [27]	UK (N)	Self-administered for Members of Parliament; “interviewer” administered for health professionals and general consumers	Not indicated	Total: 91 (54); Health Policy Makers (includes Members of Parliament): 36%; Health professionals: 21%; General consumers: 43%	Not indicated	Not indicated
Consensus Action on Salt and Health (2010) [28]	UK (Y) ^¶^	Face-to-face; interviewer administered	Not indicated	2063 (NA)	≥16	Not indicated
Health Canada (2009) [29]	Canada (Y) ^¶^	Telephone survey	Self-developed	1216 (NA)	≥18	Not indicated
Grimes *et al.*, (2009) [30]	Melbourne, Australia (N)	Intercept survey	Self-developed. Used some items from past surveys	474 (65)	≥18	75
International Food Information Council (2011) [31]	USA (Y) ^¶^	Online survey	Not indicated	2009: 1003 (51) 2011: 1003 (50)	≥18	2009: 53 2011: 55
Kim et al., (2012) [32]	Raleigh/Durham, N.C., USA (N)	Online survey	Not indicated	489 (75)	18–65	54
Kim *et al.*, (2012) [25]	Seoul (Korea) ^§^	Online survey	Past survey [32]	257 (100)	25–49	89.5
Land *et al.*, (2014) [33]	Lithgow, Australia (N) ^¶^	Face-to-face; interviewer-administered	Adapted from the World Health Organization/Pan American Health Organization protocol for population level sodium determination [34]	419 (55)	20–88 (55.4)	37
Marakis *et al.*, (2014) [35]	Greece	Telephone survey	Self-developed and circulated to experts for comments, pilot tested	3609 (52)	25–90	33.9
Marshall *et al.*, (2007) [36]	Scotland, UK (N)	Not indicated	Self-developed	118 (100)	≥18	Not indicated
Neale *et al.*, (1993) [22]	Nottingham, UK (N)	Intercept survey; interviewer administered	Not indicated	160	Not clearly indicated	Not indicated
Newson *et al.*, (2013) [20]	Germany, India, Austria, USA, Hungary, China, South Africa, and Brazil (Y) ^§^	Online survey	Self-developed	Total: 6987 (50); Germany/Austria: 998; USA: 1000; Hungary: 996; India: 1000; China: 999; South Africa: 996; Brazil: 998	18–65 (39.7)	Not indicated
Papadakis *et al.*, (2010) [37]	Ontario, Canada (N) *	Telephone survey	Self-developed based on several health promotion theories	3130 (63.9)	35–50 (44.8)	76.2
Sarmugam *et al.*, (2013) [38]	Australia (N)	Online survey	Used psychometrically validated questionnaire [39]	530 (58.3)	≥18 (49.2)	59.1
Sarmugam *et al.*, (2014) [39]	Australia (N)	Online survey	Self-developed with reference to items from past surveys. Questionnaire was tested for construct validity and internal consistencies	Total: 109 (93.1); Dietitians/nutritionists: 41 (94.9); Dietetics/nutrition students: 32 (96.8); Lay people: 36 (87.1)	≥18	80.5
Webster *et al.*, (2010) [40]	Australia (N) ^¶,§^	Online survey	Not indicated	1084 (52)	14–85	54
Welsh *et al.*, (2014) [41]	Shawnee County, Kansas, USA (N) *	Telephone survey	Adapted from several national surveys and state health surveys	834 (52.3)	≥18	25.6
Wyllie *et al.*, (2011) [42]	New Zealand (Y) ^¶^	Telephone survey	Self-developed. Questions were made similar to past surveys	1000 (52)	≥18	Not indicated					
Zhang *et al.*, (2013) [21]	Shandong Province, China (N) *	Face-to-face, interviewer-administered	Not indicated	15,350 (49.9)	18–69	7.8					

^¶^ Data were weighted during analysis to reflect country census; ^§^ Indicated data may not be generalized to whole population; ^†^ Based on unweighted data; * Data were weighted to represent study population.

### 3.2. Declarative Salt Knowledge

#### 3.2.1. Awareness of Salt Intake Recommendations

Fifteen studies examined participants’ awareness of salt intake recommendations (Table 2). Despite the differences in education levels and the countries where the studies were conducted, the majority (at least 70%) of the participants in 13 studies were unable to correctly recall or identify the recommended amount of salt intake. In four of these studies, fewer than 10% were able to identify the recommended amount of salt intake [19,24,30,31].

#### 3.2.2. Understanding of Salt and Sodium

Nine studies examined whether participants understood the meaning of “salt” and “sodium”. Fewer than half of the participants in six studies correctly identified that salt contains sodium. Two studies with higher proportions of respondents who were able to do so included groups of health care professionals [27,39].

#### 3.2.3. Knowledge of the Salt Content of Commonly Consumed Foods

In those studies that examined the perceived salt content of foods, more than half of the respondents were able to identify high salt items such as bacon and chips. Nine studies examined whether respondents were aware that processed foods are the main sources of salt in their diet. In seven of these studies, more than 70% of the respondents indicated that they are. However, two large-scale surveys conducted in the UK and the USA reported that participants tended to identify foods with the highest amounts of sodium per serving as those that contribute most salt to the diet. In both surveys, fewer than 15% of the participants could correctly identify bread and cereals as the major contributors of salt to British and American diets [28,31].

#### 3.2.4. Knowledge of Diet-Disease Relationships

Almost all studies found that at least 80% of the participants were aware of the relationships between high salt intake and hypertension. However, awareness of other cardiovascular diseases such as stroke, heart diseases or heart attacks was lower (though in the majority of studies awareness was above 60%). Fewer than half of the participants were able to correctly identify the relationships between high salt intake and lesser-known conditions such as osteoporosis.

**Table 2:** Summary of findings on assessment of declarative salt knowledge.

Author (Year)	Dietary Salt Recommendation (% Correct)	Understanding of Salt and Sodium (% Correct)	Food Sources of Salt in the Diet/Salt Content of Foods	Diet-Disease Relationships ^¶^ (% Correct)
Arcand *et al.*, (2013) [23]	Recommended sodium levels (1500 mg): 15.5%; Maximum sodium levels (2300 mg): 12.4%	Not applicable	Not applicable	Not applicable
Charlton *et al.*, (2010) [24]	5%	Not applicable	● 88% identified processed foods such as breads, breakfast cereals, tinned foods and takeaway foods as the major sources of salt in the diet.	● Salt worsens health: 62%
			● >80% identified salt content in foods such as bacon pizza and vegemite as high. More than 70% were able to identify salt content in fresh foods such as carrot, cooked rice and full cream milk as low. Only 26% correctly identified salt content in cornflakes.	● Hypertension: 97%
				● Heart attack: 88%
				● Stroke: 72%
Claro *et al.*, (2012) [19]	7%; No detailed information for countries were provided	Total: 75.6%; Argentina: 89%; Canada: 73.1%; Chile: 82%; Costa Rica: 63.9%; Ecuador: 70% ^§^	Not applicable	● Eating a diet high in salt can cause serious health issues (% agree)
				● Total: 88.5%; Argentina: 97.5%; Canada: 93.2%; Chile: 89%; Costa Rica: 86.9%; Ecuador: 75.6%.
Consensus Action on Salt and Health (2003) [ 27]	75% (Policy makers (MP) and health professionals (HP)). 43% general consumers (GC)	57%	● Main source of salt is in processed foods: 100% (MP), 89% (HP).	● Hypertension: 98% (MP), 100% (HI), 97% (GC).
				● Stroke: 67% (MP), 58% (HI), 51% (GC).
			● 61% were unaware of salt content hidden in foods like cornflakes.	● Kidney disease: 42% (MP), 21% (HI), 18% (GC).
				● Osteoporosis: 11% (total participants).
Consensus Action on Salt and Health (2010) [ 28]	Not applicable	Not applicable	The foods most commonly mentioned as the foods that contribute most salt to the UK diet (from a list of 10 foods) were crisps and snacks (73%), ready meals (65%) and meat products (36%). However, only meat products were actually in the top three. Only 13% mentioned bread, and 12% mentioned breakfast cereal.	● 92% were aware that salt can damage their health
				● Hypertension: 69%
				● Stroke: 34%
				● Heart disease: 61%
				● Kidney disease: 27%
				● Osteoporosis: 4%
Health Canada (2009) [ 29]	75% of respondents provided estimates that are within the range of adequate daily intake (0–1500 mg).	8%	● 72% believed processed foods are the single largest source of salt in Canadian diet.	● 92% agree around 80 per cent of salt in the average Canadians’ diet comes from processed food.
			● Others believed the following were the sources of where most salt in Canadian diet comes from: Salt added during cooking (4%), salt added at the table (9%), restaurant foods (13%).	● Hypertension: 96%
			● The majority identified the following foods as high in salt: processed meats (90%), canned soups (77%), pickled foods (74%) and frozen dinners (74%) and the following foods as low in salt: fresh meat or fish (70%), fresh vegetables (90%), whole wheat breads (48%) and the following as foods with moderate amount of salt: cheese (47%), canned tuna (42%).	● Stroke: 85%
				● Heart disease: 92%
				● Osteoporosis: 54%
Grimes *et al.*, (2009) [30]	5%	35%	Not applicable	● Hypertension: >88%
				● Stroke: about 60%
				● Kidney disease: about 50%
				● Osteoporosis: about 10%
International Food Information Council (2011) [ 31]	8%	Not applicable	● Most participants believed most salt in their diet comes from packaged and processed foods (2009: 45%; 2011: 43%). Others believed the following were the sources of where most salt in their diet comes from: Salt added during cooking (2009: 13%; 2011: 14%), salt added while eating (2009: 14%; 2011: 14%), restaurant foods (2009: 13%; 2011: 17%) and naturally occurring salt in foods (2009: 15%; 2011: 12%).	Salt is perceived as one of the greatest factors that impact hypertension (26%).
			● The following items were identified correctly as foods that are high in salt per serving: chips and crackers (55%), lunch meat and hot dogs (54%), canned soups (50%), condiments (30%), frozen meals (29%) and pizza (17%). *Note: data were given only for 2011.*	
			● Participants believed the following foods contribute the most amount of salt to their personal diet: snacks like chips and crackers (52%), lunch meat, hot dogs (36%), canned soups (32%). Less than 10% could identify cereal and grain products as the greatest contributors to salt intakes in the *US. Note: data were given only for 2011.*	
Kim *et al.*, (2012) [32]	Not applicable	Not applicable	● More than 90% correctly identified 7 out of 8 items low in salt (apples, fresh green beans, cookies, chocolate, Jello-O, yoghurt and steamed fish) correctly as low in salt.	● Relationships between disease and salt/sodium
				● Hypertension: 96.1/97.1%
				● Heart disease: 12.3/9.8%
			● More than 70% correctly identified 11 out of 15 items as high in salt (examples: potato chips, ham, pickles); 3 high salt items least identified as high salt were processed cheese, cottage cheese and cheddar cheese. Participants were also unlikely to think of these foods as salty.	● High cholesterol: 39.1/30.7%
				● Stroke: 82.2/86.3%
				● Kidney disease: 67.9/68.5%
				● Bone health: 30.9/36.6%
Kim *et al.*, (2014) [25]	Not applicable	Not applicable	● More than 70% correctly identified 10 out of 17 items low in salt (candy, jelly, apple, cabbage, broccoli, yoghurt, milk, egg, ice-cream, and raw fish) correctly as low in salt.	● Relationships between disease and salt/sodium
				● Hypertension: 99.6/99.2%
			● More than 80% correctly identified 8 out of 10 items as high in salt (examples: potato chips, ham, cheese, pizza, pasta); although more than 90% associated instant noodles as salty, only 60% correctly identified instant noodles as high in salt.	● Heart disease: 98.4/99.2%
				● High cholesterol: 12.8/9.7%
				● Kidney disease: 98.4/98.8%.
Land *et al.*, (2014) [33]	18%	Not applicable	Not applicable	● A diet high in salt can cause serious health problems (95%).
				● Hypertension: 81%.
Marakis *et al.*, (2014) [35]	11.1%	34%	Main source of salt in diet: salt in cooking (38%), bread (3%), meat and sausages (20%).	Diet high in salt could cause serious health problems (95%)
				● Hypertension: 69%
				● Kidney stones: 59%
				● Osteoporosis: 31%
Marshall *et al.*, (2007) [36]	28%	32%	67% agreed with the statement “65%–70% of salt intake comes from processed foods”.	89% agreed eating salt raises blood pressure, 25% agreed salt plays a part in osteoporosis.
Neale *et al.*, (1993) [22]	Not applicable	Not applicable	Knowledge on salt content of ten selected foods (cornflakes, tomato ketchup, soft margarine, cheddar cheese, white bread, baked beans, milk chocolate, cod, tomato and apple) was presented as score. Possible score (0–20). Average score was 10.34. This was only 3.1 higher than the value expected by chance (7.2). Indicates poor knowledge.	Salt is detrimental to health (83.8%).
Newson *et al.*, (2013) [20]	All countries: 13%; Germany/Austria: 10%; USA: 3%; Hungary: 9%; India: 12%; China: 34%; South Africa: 10%; Brazil: 12%.	Not applicable	Main source of salt in diet (% across all countries): salt added during food preparation (42%), salt from salt containing foods (30%), salt added at the table (14%), salt from out of home foods (14%). This pattern was evident in India, China and Brazil and different in Germany/Austria, USA, Hungary and South Africa where processed foods was thought to be the main source of salt intake, and salt added during preparation was the second most rated main source of salt (no details on percentage for each country).	“Salt in my food increases blood pressure” (scale 1–7: strongly disagree–strongly agree). All countries: 5.1 ± 1.8; Germany/Austria: 4.5 ± 1.7; USA: 5.1 ± 1.6; Hungary: 4.8 ± 1.9; India: 5.0 ± 1.9; China: 5.3 ± 1.5; South Africa: 5.1 ± 1.7; Brazil: 5.6 ± 1.9.
Papadakis *et al.*, (2010) [37]	Not applicable	Not applicable	● 89.6% believed processed foods are main sources of salt in the diet. ● More than 80% were able to identify 8 out of 18 items as high salt foods (sausages and hotdogs, luncheon meat, canned meats, frozen dinner, salted snacks, bacon, canned entrees, canned vegetables or vegetable juice and soy sauce), 3 out of 18 food items with high salt that were correctly identified by less than 50% of the respondents as high in salt were processed cheese, hamburgers, mustard and ketchup.	NA
Sarmugam *et al.*, (2014) [39]	20%	70%	60% correctly identified “bread is one of the main sources of salt in Australians’ diets”, and 50% or less were able to identify the salt content in white bread and cornflakes.	● Hypertension: 100%
				● Stroke: 100%
			● More than 70% were able to identify items which had high salt content such as bacon, processed cheese, and low salt content such as rice and mixed vegetables.	● Kidney disease: 90%
				● Osteoporosis: 40%
Webster *et al.*, (2010) [40]	14%	<50% (no detailed results were presented).	● Processed foods are main sources of salt in the diet: about 75%.	● Salt worsens health: 67%
				● Hypertension : 87%
			● On average, participants were able to correctly classify 10 common foods as high, medium or low in salt content two thirds of the time (no detailed results were presented).	● Stroke: 77%
				● Heart attack: 75%
				● Kidney disease: 44%
Welsh *et al.*, (2014) [41]	Not applicable	Not applicable	● 83.2% strongly agreed or agreed that most of the salt in the diet comes from packaged, processed, store-bought, and restaurant foods.	Hypertension: 93%
			● 65.2% strongly agreed or agreed that only a small amount of the salt in their diet comes from salt added during cooking and from salt added at the table.	
Wyllie *et al.*, (2011) [42]	25%	36%	Main source of salt in diet: salt in processed foods such as breads, breakfast cereals, tinned foods and takeaways (77%)	● Hypertension: 83%
				● Heart attack: 85%
				● Stroke: 72%
				● Kidney disease: 58%
				● Osteoporosis: 18%
Zhang *et al.*, (2013) [21]	29.3% (urban sample), 19.2% (rural sample)	Not applicable	Not applicable	● Hypertension: 60.3% (urban)
				● Hypertension: 49.0% (rural)

^¶^ For ease of comparison, only commonly assessed diseases across studies are listed here; ^§^ Information presented as “know the difference between salt and sodium”, and may not be necessarily correct.

### 3.3. Procedural Knowledge

Only three studies reported assessment of procedural knowledge (Table 3). All three studies assessed skills and comprehension using food labels to select foods with lower salt content, however only two reported findings of procedural knowledge [30,39]. Both studies found that a majority of individuals were able to use nutrition information panel to select food products with lower salt content. However, Grimes *et al.* [30] found less than 50% of participants were able to use the percentage daily intake (%DI) system to rank food with highest to lowest salt content.

**Table 3:** Summary of findings on assessment of procedural salt knowledge.

Author (Year)	Procedural Knowledge
Grimes *et al.*, (2009) [30]	Two items were used to assess comprehension of nutrition information regarding salt on food labels. 42% were able to rank three types of bread from low to highest salt content using the nutrition information panel. 84% were able to correctly identify breakfast cereals with lower salt content using percentage of daily intake (%DI).
Sarmugam *et al.*, (2013) [38]	Two items were used to assess ability to use a food label to identify pasta sauce with lower salt content and breakfast cereal using front-of-pack logo (Tick logo). The correct answers were scored and the sum of scores was used to examine the relationships between procedural knowledge and discretionary salt use.
Sarmugam *et al.*, (2014) [39]	Two items were used to assess ability to use a food label to identify pasta sauce with lower salt content and breakfast cereal using positive front-of-pack logo (Tick logo). More than 80% were able to identify pasta sauce with higher salt content, and use the Australian Heart Foundation Tick Logo.

### 3.4. Relationships between Salt Knowledge and Salt Intake/Salt-Related Dietary Practices

Fifteen studies reported outcomes of dietary salt intake or salt-related dietary practices such as discretionary salt use, consumption of high salt foods or purchasing of reduced or low salt foods (Table 4). Four studies included measures taken by participants who were trying to limit their sodium intake. Four studies reported the mean salt intake of the study population. Of these, two studies used 24-h urinary salt excretion to measure dietary salt intake [24,33]. Mean salt intakes reported by all four studies were above the WHO’s recommended salt intake of 5 g per day. Eight studies reported use of discretionary salt use. Although, the results of all eight studies cannot be compared due to use of different measures, several studies [20,33,35,38,40] consistently reported that about 20% of the study samples usually or always used discretionary salt (with the exception of Indian samples in the study conducted by Newson *et al.* [20].

Five studies analyzed the relationships between salt knowledge or beliefs with dietary salt intake [33] and salt-related behavior [21,30,38,39]. With the exception of two studies [38,39], analyses of the relationships between salt knowledge and the outcomes of interest were conducted using a single knowledge or awareness item.

**Table 4:** Summary of relationships between salt knowledge and salt intake/salt-related dietary practices.

Author (Year)	Dietary Salt Intake/Salt-Related Practices Measurement	Results of Dietary Salt Intake/Salt-Related Practices	Associations between Knowledge and Dietary Salt Intake/Salt-Related Practices
Arcand *et al.*, (2013) [23]	Not applicable	● 59.3% respondents reported they were currently trying to limit their sodium intake.	Not applicable
		● 72.5% of those limiting their sodium intake avoided high-salt foods. 45.9% of those limiting their salt intake did not avoid high salt foods, but thought their salt intake was lower because they do not add salt to their food.	
Charlton *et al.*, (2010) [24]	24-h urinary Na excretion and three-day food record.	● Mean salt intake measured using 24-h Na excretion: 6.4 g/day. 65% exceeded WHO recommended maximum level of 5 g.	Not applicable
		● Add salt in cooking: 68% “sometimes”. Add salt at the table: 67.5% “sometimes”.	
		● Almost a third never used discretionary salt.	
Grimes *et al.*, (2009) [30]	Self-reported frequencies of dietary practices.	● Purchased a product labeled “reduced salt” in the past: 70%.	In comparison to those were unaware, a higher proportion of participants were aware of the risk of hypertension (66% *vs.* 73%; Pearson χ^2^ 23.12, df = 4, *p* = 0.001), and stroke (62% *vs.* 75%; Pearson χ^2^ 18.89, df = 4, *p* = 0.001) with a high salt intake reported they had previously purchased reduced salt labeled products.
Health Canada (2009) [29]	Self-reported frequencies of action taken to reduce salt intake.	68% respondents reported they take actions to control their salt intake. Among actions reported taken to reduce salt intake are:	Not applicable
		● Do not add salt when cooking (42%).	
		● Do not add salt at the table (39%).	
		● Avoid/Minimize consumption of processed foods (24%).	
		● Look at Nutrition Facts Tables on food (21%).	
		● Monitor use of salty foods (19%).	
		● Buy low salt and low sodium foods (15%).	
		● Avoid eating out (7%).	
		● Buy/cook with fresh foods (7%).	
		● Buy low salt/sodium alternatives (6%).	
		● Use spices other than salt when cooking (6%).	
International Food Information Council (2011) [31]	Self-reported frequencies of dietary practices.	● Consumed a low or reduced sodium product: 74% “yes”, 10% “no” and 16% “don’t know”.	Not applicable
		● Frequency of purchasing low or reduced sodium products: 7% “usually”, 17% “often” 56% “occasionally” and 20% “never”.	
Land *et al.*, (2014) [33]	24-h urinary salt excretion and self-reported frequencies of salt use at the table and in cooking.	● Mean 24-h urinary salt excretion: 8.8 g/day. 87% exceeded WHO recommended maximum level of 5 g.	No significant difference in urinary salt excretion between those who correctly answered the following knowledge questions and those who did not: (1) maximum amount of recommended salt intake; (2) a diet high in salt can cause serious health problems; and (3) a diet high in salt causes hypertension) before or after adjustment for age, sex, body mass index and the highest level of education.
		● Add salt at the table: 52% “rarely”, 27% “sometimes”, 21% “always”.	
		● Add salt in cooking: 54% “rarely”, 27% “sometimes”, 19% “always”.	
		63% respondents reported they take actions to control their salt intake. Among actions reported taken to reduce salt intake are:	
		● Avoid consumption of processed foods (44%).	
		● Check food labels (30%).	
		● Buy low salt alternatives (34%).	
		● Use spices (29%).	
		● Avoid eating out (20%).	
Marakis *et al.*, (2014) [35]	Self-reported frequencies of dietary practices.	● Added salt in cooking: 5.8% “never”, 9.2% “occasionally”, 72.4% “always”.	Not applicable
		● Added salt at the table: 51.8% “never”, 15.1% “occasionally”, 6.2% “always”.	
		● Read nutrition information on food packaging: 28.4% “never”, 24.8% “always”.	
		Among actions reported taken to control salt intake are:	
		● Avoid consumption of processed foods (77.6%).	
		● Remove salt from foods in brine (70.3%).	
		● Avoid eating added salt later or used table salt (48.7%).	
Neale *et al.*, (1993) [22]	Self-reported frequencies of dietary practices and food shopping behavior.	● Frequency of eating savory snacks such as crisp and salted nuts (as crude indicator of salt taste preference): 25% “once a day or more”, 12% “5 times/week”, 23% “3 times/week”, 25% “once a week”, 15% “less than once a week”.	Not applicable
		● Purchased reduced salt products in the last one month: 65.8%, between one and three months ago: 21.7%, between four and six months ago: 12.5%.	
Newson *et al.*, (2013) [20]	Semi-quantitative food frequency questionnaire (Salt FFQ).	● Average salt intake across all countries was 9.5 g/day.	Not applicable
		● Discretionary salt use: Add salt before tasting: 58% “never/rarely”, 19% “sometimes”, 22% “usually/always”. Findings consistent across countries.	
		Sources of dietary salt intake:	
		● All countries: 51% salt containing food groups (PF), 7% salt added at the table (ST); 23% salt added during cooking (SC); 17% out of home foods (OH).	
		● Germany/Austria: 63% PF, 6% ST, 17% SC, 14% OH.	
		● USA: 70% PF, 7% ST, 9% SC, 13% OH.	
		● Hungary: 56% PF, 5% ST, 23% SC, 15% OH.	
		● India: 32% PF, 10% ST, 48% SC, 10% OH.	
		● China: 48% PF, 11% ST, 27% SC, 14% OH.	
		● South Africa: 72% PF, 7% ST, 16% SC, 5% OH.	
		● Brazil: 36% PF, 5% ST, 18% SC, 41% OH.	
Papadakis *et al.*, (2010) [37]	Self-reported consumption frequencies of food items which are largest contributors to Canadian’s sodium consumption and use of salt at the table and in cooking.	● Added salt in cooking (mean frequency in the past month (±SD)): 14.3 ± 19.4 times.	Not applicable
		● Added salt at the table (mean frequency in the past month (±SD)): 11.0 ± 18.3 times.	
Sarmugam *et al.*, (2013) [38]	Self-reported frequencies of dietary practices.	● Added salt in cooking: 35.1% “never/rarely”, 29.2% “sometimes”, 23.6% “usually”, 11.1% “always”.	Bivariate analysis showed salt knowledge scores was negatively correlated with salt use (*r* = −0.17; *p* < 0.001), misconceptions were positively associated with the salt use (*r* = 0.09; *p <* 0.05). No significant association was found between procedural knowledge scores and salt use. Structural equation modeling showed a negative direct effect of declarative knowledge on salt use (*β* = −0.12, *p <* 0.01).
		● Added salt at the table: 48.1% “never/rarely”, 26.2% “sometimes”, 17.0% “usually”, 8.7% “always”.	
Sarmugam *et al.*, (2014) [39]	Self-reported frequencies of dietary practices and food shopping behavior.	Results of the frequencies of dietary practices were not reported.	There were significant associations between the total salt knowledge scores and frequent use of salt at the table (*r* = −0.197, *p <* 0.05) and consumption of fast food (*r* = −0.293, *p <* 0.01); cooking meals from scratch/fresh ingredients (*r* = 0.321, *p <* 0.01), using herbs and spices as flavoring for cooking (*r* = 0.327, *p <* 0.01); eating fast foods and looked at salt content when shopping for food (*r* = 0.400, *p <* 0.01).
Webster *et al.*, (2010) [40]	Self-reported frequencies of dietary practices.	● Added salt during cooking: 21% “often”.	Not applicable
		● Added salt at the table: 21% “often”.	
Welsh *et al.*, (2014) [41]	24-h web based dietary recall.	● Mean salt intake measured using 24-h web based dietary recall was 8.77 g/day.	Not applicable
		● Added salt very often in cooking or preparing foods in their household: 4.2%.	
		● On average, had two or more meals prepared outside of the home per week: 42.5%.	
		● Consumed processed meals at least once a day: 8.5%.	
		● Consumed salty snacks at least once a day: 9.6%.	
		● Consumed frozen entrees at least once a day: 3.0%.	
		● Consumed canned or packaged soups at least once a day: 2.3%.	
Zhang *et al.*, (2013) [21]	Self-reported frequencies of dietary practices used to control dietary salt intake.	45.6% of urban and 34.8% of rural respondents reported they had taken actions to control their salt intake. Among actions reported taken to control salt intake are:	Multiple logistic regression analysis controlled for key confounders (age, gender, marital status, residence, region, and hypertension status) found practices towards sodium reduction were more likely to be taken by those who were aware that sodium intake was associated with increased blood pressure, compared to those who were not aware (OR = 2.17, 95% CI 2.01–2.34); and know the limit of salt (OR = 2.12, 95% CI 1.95–2.31).
		● Read label for salt content: 13.9% (urban), 9.7% (rural).	
		● Used less salt when cooking: 96.2% (urban), 95.7% (rural).	
		● Added salt later or used table salt: 27.6% (urban), 19.7% (rural).	
		● Used less pickles: 54.0% (urban), 44.6% (rural).	
		● Used low sodium processed foods: 21.4% (urban), 10.4% (rural).	
		● Used less high sodium condiments: 24.9% (urban), 12.2% (rural).	
		● Used green onion or garlic to improve the taste of food when not using salt: 20.2% (urban), 8.7% (rural).	
		● Used non -sodium condiments such as vinegar: 15.1% (urban), 5.0% (rural).	

Only one study reported no significant differences in salt intake between individuals who were able to correctly identify maximum recommended salt intake and relationships between salt and health [33]. In contrast, Sarmugam *et al.* [38], found negative associations between declarative salt knowledge and discretionary salt use. However there were no significant relationships between procedural salt knowledge and discretionary salt use. Others have found positive relationships between salt knowledge and lower salt dietary practices [21,39], purchase of reduced salt food products [30] and attitudes towards salt reduction [20].

## 4. Discussion

The aim of this review was to examine current levels of salt knowledge and the association of salt knowledge with dietary salt intake and salt-related dietary practices in the healthy adult populations.

Public awareness campaigns have been one of the components of successful salt reduction initiatives [43,44,45,46]. They are one of several strategies recommended by the WHO for population-based salt reduction [47]. The WHO report also explicitly notes that planning key campaign messages requires information about current levels of salt consumption and the health knowledge of the population. Further, awareness campaigns are likely to be more effective if they include practical tips on how consumers can manage their salt intake [47]. Taken together, these recommendations show the importance of understanding the levels of declarative and procedural salt knowledge of the population. It is surprising then that there has been no well-designed study that has used a validated questionnaire to provide a comprehensive assessment of the salt knowledge of the population.

### 4.1 Current Levels of Salt Knowledge in Population

This review found that most studies did not use a validated questionnaire to measure salt knowledge. Very few studies measured the relationships between salt knowledge and salt intake or salt reduction practices and only three assessed procedural salt knowledge. Thus, it is challenging to provide a comprehensive assessment of the current levels of salt knowledge of the population. However, there is a considerable amount of similarity between the questions; probably due to the use of several key reference papers. This allows us to draw several conclusions about the current levels of salt knowledge.

#### 4.1.1. Declarative Salt Knowledge

Poor understanding of the relationships between salt and sodium was consistent across several studies. Indeed, a review of consumers’ understanding and use of nutrition labelling found the relationships between salt and sodium were least understood [48]. Although, the term “salt” is commonly used and understood by consumers, the term “sodium” is often provided on food labels. Lack of understanding of the relationships between the two terms means consumers are unlikely to be able to convert the information on food labels (expressed as sodium) into information about salt [30].

Most participants were capable of estimating the salt content of high salt (and salty) foods, such as bacon. However, many were unaware of the salt content of everyday foods such as bread and breakfast cereals despite knowing that processed foods are the main contributors of salt to the diet [24,28,39]. Fewer than 10% of Americans sampled could identify cereal and grain products (e.g., ready-to-eat cereals and bread), as the greatest contributors to sodium intakes in the USA [31]. Consumers wrongly believed that the foods with highest amounts of sodium per serving such as salted snacks and processed meats contribute the most sodium to their diets [31]. This is probably because consumers associate the presence of sodium or salt with the salty taste of the food [32]. The review also showed that many consumers were unable to correctly identify the recommended levels of daily salt intake.

Given the lack of knowledge in these three areas, consumers are unlikely to have the ability to estimate their daily salt intake and compare their intake against salt intake recommendations. Indeed, several studies have reported that participants believed their salt intakes are equal or lower than national recommendations [21,33,40] (or were satisfactory [20]), despite the strong evidence that salt intake of most populations usually exceed the recommended levels of salt intake [7,49]. Consumers may be more likely to take action to reduce their salt intake if they perceive their salt intake exceeds the recommended level [21]. Therefore, although there is widespread awareness of the negative impact of high salt intake on health, there is a possibility that salt intake remains higher than recommended levels in part because of lack of awareness of personal salt intake.

#### 4.1.2. Procedural Salt Knowledge

Procedural knowledge is equally as important as declarative knowledge and may require greater attention to ensure behavior change [17]. However, only three studies included in this review attempted to measure procedural salt knowledge, *i.e.*, consumers’ ability to identify lower salt options through label reading. Experience from the North Karelia salt reduction initiatives show that consumers need to be taught practical skills to ensure behavior change [46]. Despite knowing that processed foods are the main sources of salt in the western diet, in several studies, most consumers claimed to limit or not add salt in food preparation, or at the table [23,29,31] as a way to control their salt intake. This suggests that awareness or knowledge of the main contributors of salt in the diet (factual knowledge) alone is insufficient. Consumers need to be equipped with practical knowledge and skills to reduce salt in their diet, *i.e.*, what can they do to reduce their salt intake and how can they do it.

#### 4.1.3. Relationships between Salt Knowledge and Salt Intake

With the exception of two studies [38,39], analyses of the relationships between salt knowledge and the outcomes of interest were conducted using a single knowledge or awareness item. Thus, the conclusions derived from such studies are unlikely to assess the true relationship of salt knowledge with behavior.

### 4.2. Limitations

This review has several limitations. It did not include studies conducted in populations such as Middle Easterners and Africans although salt intake in these populations is as high as in other parts of the world [50,51]. Because this review includes only English language publications it is possible that studies from non-English speaking populations have been excluded.

Most of the large-scale studies in this review were surveys conducted over the internet. Although, the socio-demographic profile of the samples may closely represent those of the national population (as determined by censuses), these online surveys were usually based on quota samples (essentially convenience samples), and therefore their representativeness is less certain than random probability samples.

Past research has shown women and people from higher socioeconomic strata tend to possess higher levels of nutrition knowledge [52,53]. Given the greater numbers of women and those with higher education levels in the study samples there may be a bias towards higher levels of salt knowledge in the reviewed studies.

Misconceptions tend to vary between cultures. Therefore, this review did not include studies on salt-related misconceptions although these are closely linked to knowledge and may affect salt-related dietary behaviors [38]. It is acknowledged that information on misconceptions is very useful and will assist in delivery of culturally appropriate education messages.

Finally, general nutrition knowledge is unlikely to predict a specific dietary behavior such as salt consumption [17]. Thus, studies that investigated salt knowledge as part of nutrition knowledge were excluded because these studies did not aim to specifically examine salt knowledge or awareness levels. It is possible that such information may provide further insight into the current levels or awareness of salt knowledge.

### 4.3. Recommendations for Research and Practice

The following areas were identified as gaps in the current literature on salt knowledge and should be considered in future research and public education initiatives.

- There is a need for a need for a robustly validated tool to examine salt knowledge and its impact on salt intake.
- Future salt knowledge assessment should include indices of procedural knowledge.
- Examination of the relationships between salt knowledge and salt intake requires comprehensive assessment of salt knowledge instead of reliance on single items.
- There is a need for studies in countries such as those in South East Asia, Africa and the Middle East where the majority of dietary salt comes from salt added in cooking.

## 5. Conclusions

The current review provides an overview of the salt knowledge of healthy adult populations. In general, population knowledge of salt is low. It has identified several gaps in the current literature including the need for validated comprehensive salt knowledge questionnaires (and the assessment of procedural knowledge) and the lack of high quality studies which examined the relationships between salt knowledge and dietary salt intake. Better understanding of the salt knowledge of the population will facilitate the planning and implementation of consumer education programs.
